# Non-genomic effects of PPARγ ligands: inhibition of GPVI-stimulated platelet activation

**DOI:** 10.1111/j.1538-7836.2009.03732.x

**Published:** 2010-03

**Authors:** L A Moraes, M Spyridon, W J Kaiser, C I Jones, T Sage, R E L Atherton, J M Gibbins

**Affiliations:** Institute for Cardiovascular & Metabolic Research, School of Biological Sciences, University of ReadingReading, UK

**Keywords:** glycoprotein VI, nuclear receptor, platelets, signaling

## Abstract

**Background:**

Peroxisome proliferator-activated receptor-_γ_ (PPAR_γ_) is expressed in human platelets although in the absence of genomic regulation in these cells, its functions are unclear.

**Objective:**

In the present study, we aimed to demonstrate the ability of PPAR_γ_ ligands to modulate collagen-stimulated platelet function and suppress activation of the glycoprotein VI (GPVI) signaling pathway.

**Methods:**

Washed platelets were stimulated with PPAR_γ_ ligands in the presence and absence of PPAR_γ_ antagonist GW9662 and collagen-induced aggregation was measured using optical aggregometry. Calcium levels were measured by spectrofluorimetry in Fura-2AM-loaded platelets and tyrosine phosphorylation levels of receptor-proximal components of the GPVI signaling pathway were measured using immunoblot analysis. The role of PPAR_γ_ agonists in thrombus formation was assessed using an *in vitro* model of thrombus formation under arterial flow conditions.

**Results:**

PPAR_γ_ ligands inhibited collagen-stimulated platelet aggregation that was accompanied by a reduction in intracellular calcium mobilization and P-selectin exposure. PPAR_γ_ ligands inhibited thrombus formation under arterial flow conditions. The incorporation of GW9662 reversed the inhibitory actions of PPAR_γ_ agonists, implicating PPAR_γ_ in the effects observed. Furthermore, PPAR_γ_ ligands were found to inhibit tyrosine phosphorylation levels of multiple components of the GPVI signaling pathway. PPAR_γ_ was found to associate with Syk and LAT after platelet activation. This association was prevented by PPAR_γ_ agonists, indicating a potential mechanism for PPAR_γ_ function in collagen-stimulated platelet activation. *Conclusions:* PPAR_γ_ agonists inhibit the activation of collagen-stimulation of platelet function through modulation of early GPVI signalling.

## Introduction

Diabetes mellitus is a major risk factor for vascular diseases and is associated with atherosclerosis and thrombotic complications [1]. Platelets play an important role in hemostasis and thrombosis, and are becoming increasingly implicated in inflammation and host defense mechanisms contributing to the pathogenesis and progression of the vascular complications of diabetes mellitus [2,3]. When blood vessels become damaged this results in the local exposure, generation or release of factors such as collagen and thrombin that trigger the function of platelets, initiating the hemostatic process. Platelet activation is associated with signaling that results in shape change and spreading, secretion and the release of multiple prothrombotic factors, and through the binding of plasma fibrinogen and von Willebrand factor (VWF) to integrin α_IIb_β_3_, this leads to the formation of a stable platelet thrombus [2,4,5].

Collagen binding to the platelet receptor glycoprotein VI (GPVI) results in clustering thereby triggering the tyrosine phosphorylation of the associated transmembrane protein, the Fc receptor γ-chain by the Src-family kinases Lyn and Fyn [6,7]. This results in the binding of the tyrosine kinase Syk, which becomes tyrosine phosphorylated and activated, leading to the tyrosine phosphorylation of the transmembrane adaptor protein linker for activation of T-cells (LAT). LAT forms a platform for the assembly of a signaling complex that includes phospholipase C_γ_2 (PLC_γ_2) which in turn becomes tyrosine phosphorylated. Phosphoinositide 3-kinase (PI3-K) is also recruited and through the generation of phosphatidylinositol (3, 4, 5)-trisphosphate, influences the recruitment and activation of phospholipase C_γ_2 (PLC_γ_2), which liberates the second messengers 1,2-diacylglycerol and inositol 1,4,5-trisphosphate. The formation of these molecules is responsible for the mobilization of calcium from intracellular stores and activation of isoforms of protein kinase C (PKC) leading to secretion and aggregation. PI3-K activity results in the regulation of protein kinase B (PKB), which is important for platelet function and thrombus formation [2,8,9].

The peroxisome proliferator-activated receptors (PPARs) consist of a family of three nuclear receptor isoforms (α, β/δ, and γ) that heterodimerize with the retinoic X receptor (RXR) and then modulate transcription of target genes [10]. PPARs play important roles in the regulation of metabolic pathways, including lipid biosynthesis and glucose metabolism [10,11]. This and implicated roles in cell differentiation, proliferation and inflammation have led to the hypothesis that the actions of PPARs may be associated with the prevention of cardiovascular complications [10–12]. Although platelets lack a nucleus, we and others have reported that they express a number of transcription factors including the steroid/nuclear receptors such as PPAR_γ_, PPAR_β/δ_, the glucocorticoid receptor (GR), oestrogen receptor (ER), retinoic X receptor (RXR) and NF-κB [13–19]. While steroid/nuclear receptors are recognized for their role in gene regulation, increasing evidence supports non-genomic actions of these receptors [20,21]. These studies have demonstrated that steroid hormones can induce rapid non-genomic modulation of cell function, although mechanisms have not been established for the non-genomic actions of the majority of these receptors.

The synthetic and clinically used drug rosiglitazone and the endogenous prostaglandin 15-deoxy-Δ^12,14^-prostaglandin J_2_ (15d-PGJ_2_) are ligands of PPAR_γ_ [10]. Rosiglitazone is a member of the thiazolidinedione (TZD) family used to treat type 2 diabetes mellitus that effectively lowers blood glucose levels although improving sensitivity to insulin [22,23]. Several clinical studies have demonstrated that the treatment of diabetic patients with thiazolidinediones exerts a cardioprotective effect, indicated by a reduction in the risk of myocardial infarction in diabetic patients with an acute coronary syndrome [24–26].

In the present study, we investigated the effects of PPAR_γ_ agonists, 15d-PGJ_2_ and rosiglitazone on collagen-stimulated platelet activation, signaling and on thrombus formation. We demonstrate that PPAR_γ_ ligands modulate the activity of the GPVI collagen receptor-stimulated signaling pathway resulting in reduced levels of platelet activation, aggregation and thrombus formation under arterial flow conditions.

## Materials and methods

### Reagents

15d-PGJ_2_, SQ29548 and GW-9662 were purchased from Biomol (Affinity Research Products, Exeter, UK). Rosiglitazone was from Cayman Chemical (Alexis Corporation, Nottingham, UK). Horm-Chemie collagen was from Nycomed (Munich, Germany) and collagen-related peptide (CRP) from Professor Richard Farndale (University of Cambridge, UK). Anti-Syk (N-19, LR), anti-PPARγ (E8), anti-LAT, anti-PLC_γ_2 antibodies and protein A/G agarose were purchased from Santa Cruz Biotechnology (Autogen Bioclear UK). Anti-Akt/PKBα was purchased from Upstate Biotechnology (Dundee, Scotland). PE-Cy5 labeled anti-CD62P(P-selectin) was obtained from BD Biosciences (Oxford, UK) and MRS2179, Fura-2 AM and dimethylsulfoxide (DMSO) were from Sigma (Poole, UK). All other reagents were from previously described sources [27,28]. PECAM-1 knockout mice were provided by Professor T. Mak (University of Toronto, ON, Canada). All protocols involving the use of animals were approved by University of Reading Ethical Review Panel and authorized by a Home Office licence.

### Human platelet aggregation assay

Washed platelets were prepared from fresh blood obtained from aspirin-free donors by differential centrifugation and aggregation measured by optical aggregometry (Chrono-log Corp., Havertown, PA, USA) as described previously [29]. Informed consent from human subjects was obtained and procedures approved by the University of Reading Research Ethics Committee.

### Mouse platelet aggregation assay

Platelets were isolated from mouse blood (PECAM-1-deficient mice on a C57/Bl6 genetic background and matched C57/Bl6 controls), by cardiac puncture after termination, washed, counted using a Z2 coulter counter (Beckman Coulter, Hialeah, FL, USA) and aggregation assays performed at a density of 4 × 10^8^ cells mL^−1^ by optical aggregometry as described previously [30–32].

### Immunoprecipitation and immunoblotting

For protein precipitation assays, platelets were suspended at 8 × 10^8^ cells mL^−1^ in buffer containing 1 mmol l^−1^ ethylene glycol tetraacetic acid (EGTA), 10 μmol L^−1^ indomethacin and 2 U mL^−1^ apyrase to prevent platelet aggregation, release of TXA_2_ and the secondary effects of adenosine 5´-diphosphate (ADP), respectively. Immunoprecipitation, sodium dodecylsulfate-polyacrylamide gel electrophoresis (SDS-PAGE) and immunoblotting onto polyvinylidine difluoride membrane were performed using standard techniques [28,32,33]. Densitometry was performed using a Bio-Rad GS-710 calibrated densitometer and Quantity One^®^ software (Bio-Rad, Hemel Hempstead, UK). Data were normalized for protein loading established through reprobing of each blot for the protein of interest.

### Measurement of [Ca^2+^]_i_ by spectrofluorimetry

Mobilization of calcium from intracellular stores was measured in platelets pre-loaded with the fluorescent dye FURA-2AM as described previously [27,33]. Platelets (2 × 10^8^ cell mL^−1^) were incubated with PPAR_γ_ agonist or vehicle [DMSO 0.1% (v/v)] for 3 min and then stimulated with collagen (1.0 μg mL^−1^) in a luminescence spectrophotometer (LS-50B; Perkin Elmer, Beaconsfield, UK). The ratio of emission values (excitation:340/380 nm) was calculated and converted to calcium concentration using FLWinLab software (Perkin Elmer).

### α-granule secretion

To measure α-granule secretion, surface exposure of P-selectin was assessed in whole blood by flow cytometry as reported previously [34]. In these assays the GPVI-selective agonist CRP was utilized to avoid technical issues encountered with collagen because of integrin α2β1-dependent adhesion to collagen fibrils.

### Thrombus formation in vitro

Whole fresh citrated blood was incubated with the lipophilic dye 3,3′-dihexyloxacarbocyanine iodide (DIOC_6_) and perfused through collagen-coated (100 μg mL^−1^) micro-capillaries at a shear rate of 1000 s^−1^ in the presence of PPAR_γ_ agonists 15d-PGJ_2_, rosiglitazone or vehicle control. Thrombi were subsequently visualized using a Leica DMIRE2 inverted confocal microscopy (using N PLANL 20×/0.4 objective lens with 0–2 mm correction) and thrombus volume calculated from Z series images captured using TCS SP2 software (Leica, UK), as previously reported [31,32].

### Statistical analysis

Aggregation traces are representative of at least three separate experiments from different donors. Numerical data are presented as mean ± SEM and statistical significance analyzed using the *t-*test.

## Results

### PPAR_γ_ agonists 15d-PGJ_2_ and rosiglitazone inhibit collagen-stimulated platelet aggregation

To determine if the natural PPAR_γ_ agonist, 15d-PGJ_2_ and rosiglitazone modulate platelet activation by the primary platelet agonist collagen, platelets were incubated with increasing concentrations of 15d-PGJ_2_, rosiglitazone (1, 3, 10 and 20 μmol L^−1^) or vehicle [DMSO 0.1% (v/v)] for 3, 15 or 20 min prior to stimulation with collagen (1 μg mL^−1^) for 90 s.

**Fig. 1:**
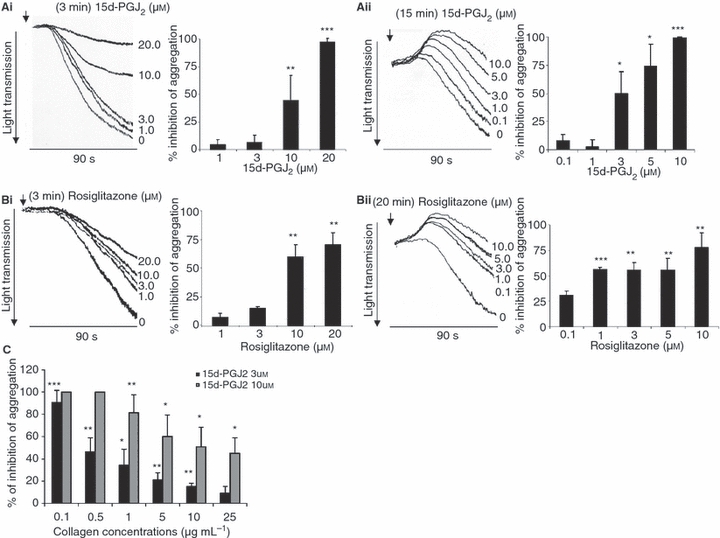
Stimulation of peroxisome proliferator-activated receptor-_γ_ (PPAR_γ_) results in diminished platelet aggregation. Washed human platelets were treated for 3, 15, or 20 min with increasing concentrations of PPAR_γ_ agonists: (Ai–ii) 15d-PGJ_2_, (Bi–ii) rosiglitazone, prior stimulation for 90 s with collagen (arrow: 1.0 μg mL^−1^) and aggregation measured at 37 °C under constant stirring conditions. Platelets were incubated with 15d-PGJ_2_ (3 and 10 μmol L^−1^) for 15 min before stimulation with increasing concentrations of collagen (0.1–25.0 μg mL^−1^) and aggregation measured (C). Numerical data represent the percentage of inhibition compared with control, mean ± SEM (*n* = 4), *t-*test *P ≤ 0.05, **P ≤ 0.01 and ***P ≤ 0.001.

Platelet aggregation in response to collagen was found to be inhibited in a concentration-dependent manner by each of the PPAR_γ_ agonists 15d-PGJ_2_ (Fig. 1Ai–ii) and rosiglitazone (Fig. 1Bi–ii). Aggregation assays performed for up to 5 min duration confirmed this effect to be inhibition rather than delay in aggregation (Fig. S1). The extent of inhibition was found to be dependent of the time of incubation with PPAR_γ_ agonists, suggesting that some differences in apparent potency may be as a result of the differential ability to cross the plasma membrane. Incubation for 15 min with 15d-PGJ_2_ (Fig. 1Aii) or 20 min with rosiglitazone (Fig. 1Bii) enabled complete inhibition of aggregation at a concentration of 10 μmol L^−1^. The effect of 15d-PGJ_2_ (3 and 10 μmol L^−1^) on platelet aggregation induced by a range of collagen concentrations (0.1, 0.5, 1, 5, 10 and 25 μg mL^−1^) was also examined. Levels of inhibition became reduced significantly with increasing concentrations of collagen. Inhibition was, however, maintained at higher concentrations of collagen. Incubation with 15d-PGJ_2_ (10 μmol L^−1^) for 15 min resulted in a significant inhibition of platelet aggregation in response to high concentrations of collagen (10–25 μg mL^−1^) (Fig. 1C).

**Fig. 2:**
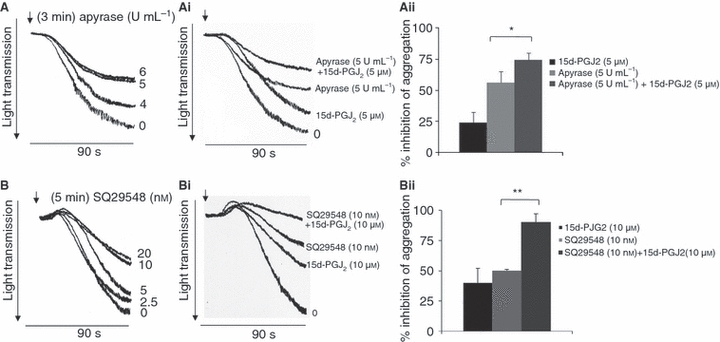
Inhibition of collagen-stimulated aggregation by peroxisome proliferator-activated receptor-_γ_ (PPAR_γ_) ligands is not dependent on inhibition of adenosine 5´-diphosphate (ADP) or TxA_2_-stimulated effects. Platelets were incubated with increasing concentrations of apyrase prior to stimulation for 90 s with collagen (arrow: 10.0 μg mL^−1^) (Ai) or 15d-PGJ_2_ (5 μmol L^−1^) plus Apyrase (5 U mL^−1^) prior to stimulation for 90 s with collagen (Aii–iii). Platelets were incubated for 5 min with increasing concentrations of SQ29548 (Bi) or 15d- PGJ_2_ (10 μmol L^−1^) plus SQ29548 (10 nmol l^−1^) (Bii–iii) prior to stimulation for 90 s with collagen (2.5 μg mL^−1^). Aggregation was measured at 37 °C under constant stirring conditions. Numerical data represent percentage of inhibition compared with control, mean ± SEM (*n* = 3), *t-*test *P ≤ 0.05, **P ≤ 0.01.

The platelet response to collagen is partially dependent on the release of secondary agonists, such as ADP and TxA_2_. Furthermore, PPAR_γ_ agonists have been previously reported to inhibit platelet aggregation induced by ADP [13]. To examine whether the inhibitory effects of PPAR_γ_ agonists on collagen-stimulated aggregation was because of their ability to inhibit the actions of TxA_2_ and ADP secreted after stimulation with collagen, the thromboxane receptor (TPα/TPβ) antagonist SQ29548 and apyrase were used. Figure 2Ai demonstrates the ability of apyrase to partially inhibit the level of collagen- (10.0 μg mL^−1^) stimulated platelet aggregation. At this concentration of collagen a maximal level of inhibition was achieved by 5 U mL^−1^ apyrase. In the presence of apyrase (5 U mL^−1^), the PPAR_γ_ agonist 15d-PGJ_2_ increased the inhibition of platelet aggregation, suggesting that the effects of PPAR_γ_ agonists on collagen-stimulated aggregation may not be explained through inhibition of ADP signaling alone (Fig. 2Aii–iii). Similar data were obtained using the P2Y_1_ antagonist MRS2179 (Fig. S2). The TxA_2_ antagonist SQ29548 partially inhibited collagen-(2.5 μg mL^−1^) stimulated platelet aggregation; a maximal level of inhibition was achieved by 10 nmol l^−1^ SQ29548 (Fig. 2Bi). In the presence of 10 nmol l^−1^ SQ29548, the PPAR_γ_ agonist 15d-PGJ_2_ enhanced inhibition of platelet aggregation, suggesting that the effects of PPAR_γ_ agonists on collagen-stimulated aggregation, may not be because of attenutation of TxA_2_ signaling alone (Fig. 2Bii–iii).

### 15d-PGJ_2_ and rosiglitazone signal through PPAR_γ_ on platelets

**Fig. 3:**
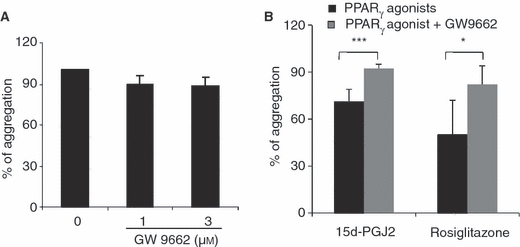
Peroxisome proliferator-activated receptor-_γ_ (PPAR_γ_) ligands 15d-PGJ2 and rosiglitazone signal through PPAR_γ_ on platelets. Washed human platelets were treated for 5 min with (A) PPAR_γ_ antagonist GW9662 (1, 3 μmol L^−1^) or (B) GW9662 (1 μmol L^−1^) followed by incubation for 15 min with PPAR_γ_ ligands 15d-PGJ2 or rosiglitazone (3 μmol L^−1^) prior to stimulation for 90 s with collagen (1.0 μg mL^−1^) and aggregation measured at 37 °C with constant stirring. Data represents percentage of (A) aggregation and (B) recovery of aggregation compared with control. Numerical data represent, mean ± SEM (*n* = 3), *t-*test *P ≤ 0.05 and ***≤ 0.001.

To establish whether the effects of PPAR_γ_ ligands on platelets are mediated by the receptor (PPAR_γ_), similar aggregation assays were carried out in the presence of the PPAR_γ_ antagonist GW9662. Washed human platelets were treated for 5 min with PPAR_γ_ antagonist GW9662 alone or followed incubation for 15 min with PPAR_γ_ ligands 15d-PGJ_2_ or rosiglitazone prior to stimulation for 90 s with collagen (1 μg mL^−1^). The PPAR_γ_ antagonist GW9662 alone (1, 3 μmol L^−1^) did not modulate the levels of collagen-stimulated aggregation (Fig. 3A). GW9662 (1 μmol L^−1^) did, however, cause a significant suppression of the inhibition of collagen-stimulated platelet aggregation by 15d-PGJ_2_ and rosiglitazone (3 μmol L^−1^) (Fig 3B). These data indicate that the effects of 15d-PGJ_2_ and rosiglitazone are mediated, at least in part, through binding to PPAR_γ_ in platelets.

### Rosiglitazone and 15d-PGJ_2_ inhibit thrombus formation under arterial flow conditions

**Fig. 4:**
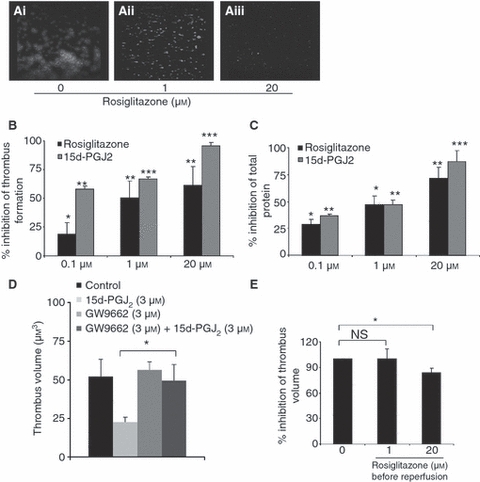
Peroxisome proliferator-activated receptor-_γ_ (PPAR_γ_) ligands inhibit thrombus formation under arterial flow conditions. Whole blood from healthy donors was incubated for 5 min with PPAR_γ_ ligands or vehicle control and perfused through collagen-coated capillaries at a shear rate of 1000 s^−1^. Composite data from Z series images were obtained by confocal microscopy (Ai–iii). Analysis of thrombus volume (B) and protein concentration (C) in the presence of increasing concentrations of PPAR_γ_ ligands was performed. The PPAR_γ_ antagonist GW96622 (3 μmol L^−1^) was incubated for 5 min prior addition of PPAR_γ_ ligand or vehicle and thrombus volume analyzed (D). To assess the impact of exposure of pre-formed thrombi to PPAR_γ_ agonist, formed thrombi were perfused at an arterial shear rate with rosiglitazone or solvent control for 5 min, and thrombus volume measured by confocal microscopy (E). Numerical data represent percentage of inhibition compared with control, mean ± SEM (*n* = 4) *t-*test *P ≤ 0.05, **P ≤ 0.01 and ***P ≤ 0.001.

The effect of 15d-PGJ_2_ and rosiglitazone on thrombus formation in whole blood was examined under arterial flow conditions *in vitro*. Whole blood was perfused through microcapillary tubes coated internally with collagen at a shear (laminar flow) rate of 1000 s^−1^ in the presence of rosiglitazone or 15d-PGJ_2_ (0.1, 1 and 20 μmol L^−1^) or vehicle [DMSO 0.1% (v/v)]. Thrombus size was calculated from the mean thrombus volume of five randomly selected fields of view. Figure 4A (i–iii) shows composite images from Z series captured and analyzed by confocal microscopy in the presence of vehicle control and rosiglitazone. Both PPAR_γ_ ligands, rosiglitazone and 15d-PGJ_2_ inhibited the thrombus formation significantly in a concentration-dependent manner, where 1 μmol L^−1^ rosiglitazone or 15d-PGJ_2_ were able to inhibit thrombus formation by 50.4 ± 14.7 % and 66.6 ± 2.7 % compared with the vehicle control (Fig. 4B). To measure thrombus formation along the whole capillary, lysis buffer was passed through each capillary and protein concentration measured as an indicator of thrombus size. This approach is important because, as a result of the fibrilar nature of the collagen used, coating of microslides may not be completely uniform. As this may influence data collected from selected fields, analysis of platelet recruitment along the entire capillary is quantitatively more reliable. Consistent with the thrombus volume data, PPAR_γ_ ligands resulted in reduced protein concentration compared with control (Fig. 4C), and no significant differences were noted between rosiglitazone and 15d-PGJ_2_ treatments. The inclusion of the PPAR_γ_ antagonist GW9662 (3 μmol L^−1^) was able to reverse the inhibitory effect of the PPAR_γ_ ligand 15d-PGJ_2_ (3 μmol L^−1^) on thrombus formation (Fig. 4D).

It is possible that PPAR_γ_ agonists may reduce thrombus stability, which may result in greater levels of embolization. To explore this, thrombi were formed under arterial flow conditions, and subsequently perfused, again at arterial shear rate, with buffer containing rosiglitazone (1, 20 μmol L^−1^) or solvent control. Thrombus volume was subsequently measured by confocal microscopy. After perfusion, a concentration of 1 μmol L^−1^ rosiglitazone caused no effect on thrombus stability, although an approximate reduction of 10% in thrombus volume was observed at 20 μmol L^−1^ (Fig. 4E).

### PPAR_γ_ ligands inhibit P-selectin exposure and collagen-stimulated mobilization of calcium

**Fig. 5:**
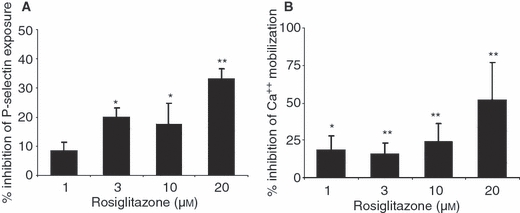
Peroxisome proliferator-activated receptor-_γ_ (PPAR_γ_) ligands inhibit P-selectin exposure and glycoprotein VI (GPVI)-stimulated mobilization of calcium from intracellular stores. (A) Whole citrated blood was pre-incubated with rosiglitazone or vehicle control for 3 min and platelet P-selectin surface exposure was measured after stimulation with collagen-related peptide (CRP) (1.0 μg mL^−1^). Data represent percentage inhibition of P-Selectin exposure compared with vehicle control [mean ± SEM (*n* = 4)]. (B) Fura-2AM-loaded platelets were incubated with rosiglitazone or vehicle control for 3 min and then stimulated with collagen (1.0 μg mL^−1^) for 200 s, and intracellular mobilization of calcium measured by spectrofluorimetry. Data shown represent percentage inhibition of peak cytoplasmic calcium concentration compared with vehicle control [mean ± SEM (*n* = 3)], *t-*test *P ≤ 0.05 and **P ≤ 0.01.

Whole citrated blood was pre-incubated with increasing concentrations of the PPAR_γ_ ligand rosiglitazone (1, 3, 10 and 20 μmol L^−1^) or vehicle [DMSO 0.1% (v/v)] for 3 min and then stimulated with GPVI-selective ligand CRP (1 μg mL^−1^) for 3 min and α-granule secretion was assessed by surface exposure of P-selectin by flow cytometry (Fig. 5A). Rosiglitazone was found to inhibit P-selectin exposure. Stimulation of the collagen receptor GPVI leads to rapid intracellular mobilization of calcium [33,35]. We therefore examined the ability of PPAR_γ_ ligands to modulate intracellular mobilization of calcium, on stimulation with collagen. Experiments were performed in the presence of 2 mmol l^−1^ EGTA to prevent extracellular calcium influx. Fura-2AM-loaded washed platelets were pre-incubated with increasing concentrations of rosiglitazone (1, 3, 10 and 20 μmol L^−1^) or vehicle [DMSO 0.1% (v/v)] for 3 min and then stimulated with collagen (1 μg mL^−1^). Rosiglitazone caused inhibition of collagen-stimulated peak calcium concentrations (Fig. 5B). It is interesting to note that some aspects of platelet function show different levels of inhibition by a given concentration of PPAR_γ_ agonist, which may also point towards mechanisms of action.

### PPAR_γ_ ligands inhibit the tyrosine phosphorylation of components of the GPVI signaling pathway

To begin to explore the mechanism through which PPAR_γ_ ligands inhibit collagen receptor-mediated signaling the effect of these ligands on the tyrosine phosphorylation of a number of receptor-proximal components of the GPVI signaling pathway was examined. Platelets were stimulated in the presence of EGTA (1 mmol l^−1^), apyrase (2 U mL^−1^) and indomethacin (10 μmol L^−1^) to prevent aggregation and ensure the study of primary signaling events. In collagen signaling studies, where non-aggregation conditions are necessary, collagen concentrations required to observe signaling were increased (25 μg mL^−1^) in order to observe tyrosine phosphorylation of components of the GPVI pathway, consistent with previous reports [32,33]. PPAR_γ_ ligand concentrations used were therefore also increased.

**Fig. 6:**
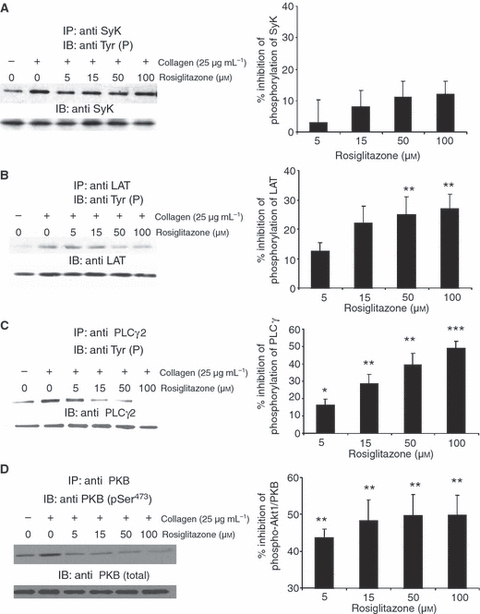
The peroxisome proliferator-activated receptor-_γ_ (PPAR_γ_) ligand rosiglitazone modulates glycoprotein VI (GPVI) signaling. Washed platelets were incubated with rosiglitazone or vehicle control for 3 min and then stimulated with collagen (25 μg mL^−1^) for 90 s. Syk (A), LAT (B), PLCγ2 (C) and PKBα/AKT were immunoprecipitated and immunoblotted to detect phosphotyrosine residues. PKBα/AKT phosphorylation (Ser^473^) was measured using a phosphospecific antibody. Equivalent protein loading was verified by reprobing for Syk (A), LAT (B), PLCγ2 (C) and PKBα/AKT (D). Densitometry analyzes were performed on replicate experiments using blood from four different donors, and data normalized for protein loading levels [mean ± SEM (*n* = 4), *t-*test *P ≤ 0.05, **P ≤ 0.01 and ***P ≤ 0.001].

The effect of rosiglitazone on collagen-stimulated tyrosine phosphorylation of Syk, LAT and PLC_γ_2 was investigated. Treatment of platelets with rosiglitazone was without a marked effect on the levels of collagen-stimulated tyrosine phosphorylation of Syk (Fig. 6A), although a trend for low-level inhibition that did not reach significance was observed. In contrast, rosiglitazone was found to cause a marked and concentration-dependent reduction in the levels of tyrosine phosphorylation of LAT (Fig. 6B) and PLC_γ_2 (Fig. 6C). The treatment of platelets with the PPAR_γ_ ligand rosiglitazone was found to result in inhibition of PI3-K activity as the levels of serine phosphorylation of a downstream marker of PI3-K signaling, Akt/PKBα, were reduced (Fig. 6D).

### PPAR_γ_ interacts with Syk and LAT upon stimulation of the GPVI pathway

**Fig. 7:**
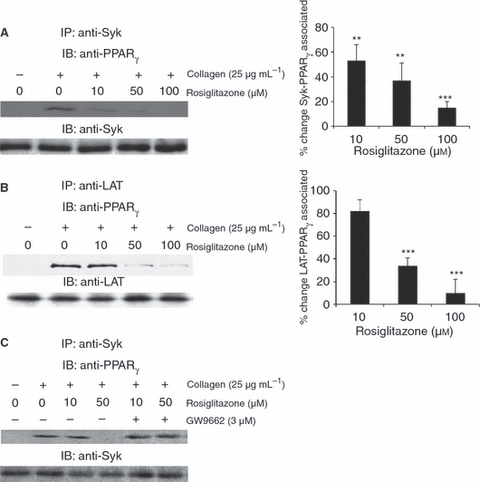
Peroxisome proliferator-activated receptor-_γ_ (PPAR_γ_) interacts with Syk and LAT upon platelet stimulation with collagen. Washed platelets were incubated with rosiglitazone or vehicle control for 15 min and then stimulated with collagen (25 μg mL^−1^) for 90 s. Syk (A) and LAT (B) were immunoprecipitated from cell lysates and immunoblotted to detect PPAR_γ_ levels. Equivalent protein loading was verified by reprobing for Syk and LAT. Densitometry analyses were performed on replicate experiments using blood from four different donors, and data normalized for protein loading levels expressed as a percentage of change in Syk-PPAR_γ_ (A) and LAT-PPAR_γ_ association (B). GW9662 (3 μmol L^−1^) was incubated with platelets for 5 min prior rosiglitazone or vehicle control for 15 min and then stimulated with collagen (25 μg mL^−1^) for 90 s (C). Blots are representative of three different experiments (*n* = 3) [mean ± SEM (*n* = 4), *t-*test **P ≤ 0.01 and ***P ≤ 0.001].

As in the presence of PPAR_γ_ agonists tyrosine phosphorylation of Syk remained unaffected, while downstream LAT phosphorylation was inhibited significantly, it was hypothesized that PPAR_γ_ may interact with Syk and/or LAT. In order to test this, Syk and LAT were immuno-precipitated from platelets treated with rosiglitazone (10–100 μmol L^−1^) for 15 min prior to their stimulation with collagen (25 μg mL^−1^) and immunoblot analyses were conducted to detect PPAR_γ_. PPAR_γ_ was found to interact with Syk and LAT when platelets were stimulated with collagen in the absence of PPAR_γ_ ligands (Fig. 7A–B). In the presence of the PPAR_γ_ ligand rosiglitazone, this interaction with both Syk and LAT was inhibited. The inhibitory effect on the PPAR_γ_–Syk interaction was prevented by the addition of the PPAR_γ_ antagonist GW9662 (3 μmol L^−1^) (Fig. 7C), indicating that this effect is PPAR_γ_ activation dependent. GW9662 also prevented rosiglitazone-dependent inhibition of PPAR_γ_–LAT interactions (data not shown).

### The inhibitory effect of PPAR_γ_ ligands on platelet function is not PECAM-1 dependent

Platelet endothelial cell adhesion molecule-1 (PECAM-1), has been reported to negatively regulate platelet function and thrombus formation [28,30,36]. Type 2 diabetes mellitus has been shown to be associated with the cleavage of platelet PECAM-1. These changes were reverted in patients treated with rosiglitazone, leading Randriamboavonjy *et al.* [37] to suggest that rosiglitazone may contribute to a decrease in the development of vascular diseases associated with type 2 diabetes mellitus through actions on PECAM-1. In order to establish if the inhibitory effect of collagen-stimulated platelet function by acute exposure to PPAR_γ_ ligands *in vitro* was dependent on PECAM-1, the effect of rosiglitazone on platelet aggregation was examined using washed platelets from PECAM-1-deficient mice.

**Fig. 8:**
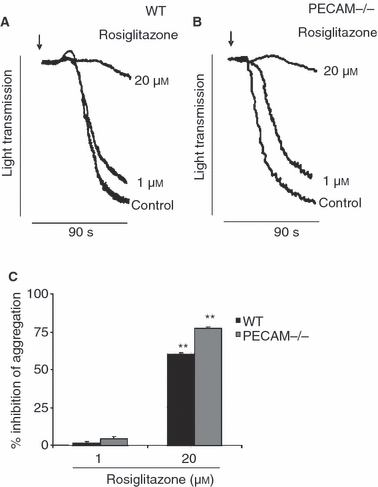
Inhibitory effect of collagen-stimulated platelet aggregation by peroxisome proliferator-activated receptor-_γ_ (PPAR_γ_) ligands is not platelet endothelial cell adhesion molecule-1 (PECAM-1) dependent. Washed platelets obtained from wild-type (WT) mice (A) and PECAM-1-deficient mice (B) were treated with PPAR_γ_ ligand rosiglitazone (1, 20 μmol L^−1^) or vehicle [DMSO 0.1% (v/v)] and stimulated with collagen (1.0 μg mL^−1^). Aggregation was measured under constant stirring conditions at 37 °C. Representative aggregation traces (A–B) and cumulative data (C) represent the percentage of inhibition compared with control. Numerical data represent, mean ± SEM (*n* = 3) *t-*test **P ≤ 0.01.

Consistent with previous reports [30,36], platelets derived from PECAM-1-deficient mice exhibit a mildly exaggerated GPVI-mediated aggregation response to collagen when compared with wild-type mouse platelets (controls Fig. 8A,B: reduced lag phase and faster initial kinetics). Collagen-stimulated platelet aggregation in wild-type and PECAM-1-deficient platelets was inhibited in the presence of PPAR_γ_ ligand rosiglitazone, when compared with the vehicle control (Fig. 8A–C). This indicates that the acute (i.e. non-genomic) inhibitory effects of rosiglitazone on platelet function are not dependent on the presence or function of PECAM-1.

## Discussion

While platelets are anucleate cells, recent reports have demonstrated that nuclear receptors such as the glucocorticoid receptor [15], RXR [17] and PPAR isoforms_γ_ [13] and β/δ [14] are expressed in these cells. Indeed, these studies have demonstrated the ability of ligands for intracellular receptors to regulate platelet function in a non-genomic fashion [13–19]. PPAR_γ_ can be activated by a number of ligands, including lipids and eicosanoids, such as 5,8,11,14-eicosatetraynoic acid and the prostanoids PGA_1_, PGA_2_, PGD_2_ and 15d-PGJ_2_, docosahexaenoic acid, linoleic acid and the synthetic anti-diabetic glitazones (e.g. rosiglitazone) [10,11].

PPAR_γ_ ligands have been reported to inhibit platelet aggregation in response to ADP that is accompanied by a reduction in markers of platelet activation such as P-selectin exposure, TXA_2_ synthesis and sCD40L release [13]. Recently, we demonstrated that RXR ligands inhibit platelet activation stimulated by ADP or the TXA_2_ mimetic U46619 and have proposed this to be mediated through suppression of Gq signaling, resulting in inhibition of mobilization of calcium from intracellular stores [17]. Although this has yet to be explored, the ability of PPAR_γ_ to interact with RXR may suggest some overlapping modes of action.

In this study, we have demonstrated that PPAR_γ_ ligands inhibit collagen-stimulated platelet aggregation, α-granule secretion and calcium mobilization. In the presence of the PPAR_γ_ antagonist GW9662, inhibition of aggregation was reversed, suggesting that this affect is at least in part, modulated by PPAR_γ_ in platelets. Failure to completely reverse inhibition indicates potential additional, and as yet uncharacterized, PPAR_γ_-independent modes of action of these ligands. Increased concentrations of rosiglitazone or 15d-PGJ_2_ were associated with more accentuated levels of shape change upon stimulation with collagen. This is likely to reflect lower levels of aggregation in this optical assay, as PPAR_γ_ agonists alone do not stimulate shape change. We cannot, however, rule out the possibility that PPAR_γ_ normally serves to inhibit shape change.

As PPAR_γ_ agonists were found to inhibit collagen-stimulated calcium mobilization, a range of signaling proteins upstream of calcium in the GPVI collagen activation pathway were examined. Rosiglitazone did not cause marked inhibition of collagen-stimulated tyrosine phosphorylation of the kinase Syk, suggesting that the activity of upstream Src-family kinases, such Fyn and Lyn, is not modulated by PPAR_γ_ ligands. This ligand was, however, found to reduce the levels of tyrosine phosphorylation of the transmembrane adapter protein LAT and thereby PLC_γ_2, which is consistent with the inhibition of calcium regulation and α-granule secretion.

The tyrosine phosphorylation of LAT results in the recruitment and activation of PI3-K, leading to the generation of 3′-phosphorylated inositol phospholipid second messengers. Rosiglitazone treatment resulted in diminished collagen-stimulated phosphorylation of Akt/PKBα, suggesting that the inhibitory effect of the PPAR_γ_ stimulation also results in suppression of PI3-K signaling. In the present study, interactions of PPAR_γ_ with Syk and LAT highlight a potential novel GPVI-dependent mechanism for PPAR_γ_ action on platelet activation. PPAR_γ_ in its inactivated state interacts with Syk and LAT (and possibly other components of the LAT signalosome). These interactions correlate with phosphorylation of Syk and LAT leading to the activation of proteins downstream within the GPVI pathway. Upon ligation of PPAR_γ_, interactions with Syk and LAT were prevented, which coincided with diminished signaling downstream resulting in a reduction in platelet activation. The addition of the antagonist GW9662 was able to prevent the inhibitory effect of PPAR_γ_ ligands on interactions between PPAR_γ_ with Syk and LAT. Taken together, this suggests that the inhibitory actions of PPAR_γ_ ligands may be mediated within the GPVI signaling pathway at the level of LAT or the LAT signalosome and that inhibition of platelets by PPAR_γ_ ligands is not because of toxic effects. Further work is required to establish whether PPAR_γ_ is recruited to a signaling protein complex with both Syk and LAT, or whether interaction with Syk and LAT occurs independently. Furthermore, whether PPAR_γ_ interactions contribute to positive signaling through the GPVI pathway remains to be established.

It has been suggested that PPAR_γ_ ligands reduce the development of atherosclerosis and myocardial ischemia–reperfusion injury through inhibition of platelet activation and intra-arterial thrombus formation in animal models [38]. In support of this notion, we have observed that PPAR_γ_ ligands rosiglitazone and 15d-PGJ_2_, inhibit thrombus formation in human whole blood on immobilized collagen under arterial flow conditions. Furthermore, perfusion with a low concentration of rosiglitazone, which is likely achievable in plasma of patients taking rosiglitazone (1 μmol L^−1^) [39], caused no effect on thrombus stability. Together this suggests that PPAR_γ_ ligands may offer beneficial clinical actions through inhibition of thrombus formation without embolization effects. Future studies using *in vivo* models of thrombosis will be required to explore this further.

Treatment with TZDs such as rosiglitazone has been reported to reduce the activity of circulating platelets in patients with coronary artery disease [40] and type 2 diabetes mellitus [41]. More recently, treatment of type 2 diabetes with rosiglitazone has been reported to cause decreases in μ-calpain activity, the restoration of platelet PECAM-1 levels and diminished platelet responsiveness to thrombin [37]. As PECAM-1 and PPAR_γ_ ligands are able to inhibit the function of platelets, and they possess similar abilities to modulate calcium mobilization in these cells, we sought to determine whether the acute, non-genomic actions of rosiglitazone may be dependent on PECAM-1 expression. Examination of PECAM-1-deficient mouse platelets revealed, however, that the inhibitory effect of collagen-stimulated platelet aggregation by PPAR_γ_ ligands is unaffected by the presence or absence of PECAM-1. The possibility still exists, however, that PPAR_γ_ and PECAM-1 share similarities in their modes of modulation of GPVI-stimulated signaling in platelets.

Clinical trials have demonstrated that the treatment of diabetic patients with TZDs exerts a cardioprotective effect as evidenced by a reduction in the risk of myocardial infarction in diabetic patients [24–26,40]. However, there are conflicting reports demonstrating that administration of PPAR_γ_ agonists may be associated with an increased incidence of congestive heart failure, myocardial infarct and death [42–44]. These latter studies were limited by a lack of access to original source data, and were insufficiently statistically powered. Better characterization of such patients is therefore needed to determine the effect of TZDs on overall cardiovascular outcome.

Our findings indicate that PPAR_γ_ ligands inhibit collagen-stimulated platelet function through modulation of signaling downstream of the collagen receptor GPVI.

## Addendum

Leanardo A. Moraes and Michael Spyridon contributed equally to this work.

## Supporting Information

**Fig. S1.** Stimulation of PPAR_γ_results in diminished platelet aggregation. Washed human plateletswere treated with increasing concentrations ofPPAR_γ_ agonist 15d-PGJ2, prior stimulation for 300s with collagen (1.0 μg mL^−1^) and aggregationmeasured at 37 °C under constant stirring conditions. Tracesare representative of three separate experiments.

**Fig. S2.** Inhibition of collagen-stimulatedaggregation by PPARγ ligands is not dependent on inhibitionof ADP effects. Washed human platelets were treated for 15 min with(A) MRS2179 or (Ai-ii) 15d-PGJ2 (5 μmol l^−1^)plus MRS2179 (5 μmol l^−1^) prior to stimulationfor 90 s with collagen (1.0 μg mL^−1^) andaggregation measured at 37 °C under constant stirringconditions. Traces are representative of three separateexperiments.
