# Supplementary material for: Non-genomic effects of PPARγ ligands: inhibition of GPVI-stimulated platelet activation
Source: J Thromb Haemost. 2010 Mar;8(3):577–87. doi: 10.1111/j.1538-7836.2009.03732.x (PMC3298645; doi:10.1111/j.1538-7836.2009.03732.x)
Supplement: Supplementary file 1 [file jth0008-0577-SD1.doc]

**Supplementary data:**

**Figure 1 – Stimulation of PPAR_γ_ results in diminished platelet aggregation.** Washed human platelets were treated with increasing concentrations of PPAR_γ_ agonist 15d-PGJ_2_, prior stimulation for 300s with collagen (1.0 µg mL^-1^) and aggregation measured at 37°C under constant stirring conditions. Traces are representative of 3 separate experiments.

**Figure 2 – Inhibition of collagen-stimulated aggregation by PPARγ ligands is not dependent on inhibition of ADP effects.** Washed human platelets were treated for 15 min with (A) MRS2179 or (Ai-ii) 15d-PGJ_2_ (5 µM) plus MRS2179 (5 µM) prior to stimulation for 90s with collagen (1.0 µg mL^-1^) and aggregation measured at 37°C under constant stirring conditions. Traces are representative of 3 separate experiments.
